# The Impact of Traditional and Virtual Microscopy on Students' Learning of Histology: A Comparative Study

**DOI:** 10.7759/cureus.87668

**Published:** 2025-07-10

**Authors:** Bishnu Rabha, Krishna K Biswas, Hari C Sarangsa, Gautam C Das, Sudipto Pal

**Affiliations:** 1 Anatomy, Silchar Medical College and Hospital, Silchar, IND; 2 Anatomy, Kokrajhar Medical College and Hospital, Kokrajhar, IND; 3 Anatomy, Tinsukia Medical College and Hospital, Tinsukia, IND

**Keywords:** digitized slide, glass slides, light microscope, microscope, questionnaire study, traditional microscopy, virtual microscopy

## Abstract

Background and objective

Traditional microscopy (TM) has been the sole method of learning histology in medical school, and it is associated with several drawbacks. Virtual microscopy (VM) is a modern technique of learning histology that allows more scope for learners to improve understanding of the subject matter and may, in this way, help to alleviate the inherent problems associated with TM. This study aimed to examine the role of VM in histology teaching and evaluate students’ perceptions regarding traditional and modern approaches to histology learning.

Methodology

The study involved 100 MBBS students: 58 (58%) males and 42 (42%) females from the 2021-22 academic year at Silchar Medical College, Silchar, of the phase I MBBS curriculum, with at least 75% attendance in histology. Repeaters and students who did not provide consent to participate in the study were excluded. Both male and female participants were equally divided by random sampling into two groups: the TM and VM groups. A one-day intervention program on histology was conducted wherein both groups underwent histology learning through a didactic lecture on the cardiovascular system by an experienced faculty, followed by respective practical sessions: the TM group handled glass slides and a light microscope, whereas the VM group utilized digitized slides and a virtual microscope. The evaluation part of the study was performed in two parts: first part included histology tests, namely, multiple choice questions (MCQs) and objective structured practical examination (OSPE); in the second part, a questionnaire with six items on TM or VM was distributed to respective groups to assess the overall acceptability of the teaching method used. To compare the effectiveness of TM and VM, differences in mean scores of students’ performance on histology tests and questionnaire responses were recorded and statistically analyzed by setting significance at p<0.05 with a confidence interval (CI) of 95%.

Results

VM students excelled in both histology tests (MCQs and OSPE), and differences in performance between the two groups were found to be statistically significant (all p<0.05). This improvement in knowledge gain and understanding may be attributed to the distinct advantages of VM as a learning tool. Differences in acceptability of either TM or VM in the respective group, calculated from the questionnaire scores obtained by students, were also in favour of VM (all p<0.001).

Conclusions

Based on our findings, VM appears to be an advanced and useful method for histology learning. Hence, integrating VM slides with TM could provide the best interface for histology learning and teaching. Most of the previous studies were merely questionnaire-based, prompting us to adopt a much more comprehensive approach by including both the questionnaire and histology test to provide newer dimensions to the notion of integrating VM with TM. Moreover, as our study is the first of its kind to be conducted in a medical college in Assam, we believe it will act as a template for many similar research studies in the future.

## Introduction

Histology is a branch of anatomy that deals with the structural organization of tissues [1]. Also known as microanatomy [2], it is one of the microscopic anatomy sciences where tissue is investigated with the help of a microscope [3]. Knowledge of histology helps in correlating the structure of a tissue with its physiology, thereby facilitating the understanding of pathological processes [1,4]. Cell biology and histology are integral parts of the undergraduate medical anatomy curriculum in many countries worldwide [5]. Traditionally, histology has been one of the major areas of laboratory-based teaching curriculum and is included in the first year of medical curriculum [2,4]. The light microscope has been the primary laboratory teaching tool for histology learning in traditional microscopy (TM) [2]. TM helps students in making independent observations by increasing their ability to explore microscope slides by panning and changing magnifications [6]. But due to a lack of microscope experience, difficulty in understanding complex and static microscopic images, and correlating those to dynamic physiological functions, students often find it hard to learn histology in laboratory sessions [7].

Moreover, a light microscope comes with a limited field of vision and magnification, which further creates difficulties in getting a better view of tissue slides. Alteration of the field is also not possible for ubiquitous observation of the particular field by students, and that poses a barrier for whole slide screening [8]. So, despite its historical significance [9], TM faces challenges like operation difficulty and suboptimal image quality [10]. Another limitation is that the teacher and student are unable to view the slides concurrently, which hinders knowledge precision [11]. To combat these problems, measures are being implemented, including liquid crystal display (LCD) projection of same slide image in practical class rooms as shown under microscope [8], use of multi-viewer microscope [11], introduction of interactive white board [12,13], giving classroom task in laboratory [14], providing both formative assessments [15], and constructive feedback to students [16]. Even after adopting corrective measures, students still find histology slides difficult to identify, despite acquiring good theoretical knowledge [17]. Furthermore, TM presents logistical issues with slide storage and accessibility that further increase the laboratory work [10]. TM not only presents difficulty of having variability between sections, affecting the successful teaching process by posing frequent confusion among students, but it also demands regular change of slides to cope with damages and fading of dyes [8]. Besides, tissue on a large scale is required to build a comprehensive medical histology slide set.

Histology teaching has been greatly influenced by newer approaches with changes in medical curriculum to keep up with the rising trends [4]. The emergence and rapid advancement of microcomputer technology have paved the way for innovative methods of presenting image-rich information, as commonly seen in the histology laboratory [2]. Modern-day technologies show immense possibilities of entirely replacing traditional light microscopes in many disciplines of health and sciences [18]. Over the last two decades, advanced technologies have transformed medical education globally [9]. One such emerging technology is virtual microscopy (VM), which provides scope for physically distant histology and histopathology education, and more importantly, it is rapidly evolving [19]. VM involves whole slide imaging (WSI) technology that allows conversion of glass slides into digitized slides to be viewed on a computer screen [20]. It uses modern scanners for digitization of glass slides to high-resolution images [21]. These digitized glass slides, also called virtual slides [2,22], can be further viewed in a web browser that closely simulates the observation of glass slides with a real microscope, by using appropriate software [23]. The disadvantages associated with TM could be overcome with the use of VM, which possesses economical and pedagogical advantages, has an established role in the medical field, and is also convenient to apply among students and teachers [24]. Prospective usefulness of VM also includes active student participation, extensive learning objective coverage, its economical nature, as well as applicability of self-directed learning [25]. Handling of high-quality images for various purposes and convenient access to the virtual slides are some of the other advantages of VM [26].

VM has become a widely used technology in medical education of histology and histopathology worldwide [27]. However, in India, most of the medical colleges still use traditional methods for teaching histology, through lectures preceding practical sessions to receive hands-on training on operating a light microscope and glass slides, while the adoption of photomicrographs, digitized slides, computer-assisted techniques or other advanced technologies are still far from being realistic options [17]. The addition of VM to the presently predominant TM will definitely help the students in the process of developing self-directed learning approaches. The present study aims to assess the significance of VM in histology teaching and evaluate students’ perception regarding traditional and modern approaches to histology learning.

## Materials and methods

This study was conducted in the Department of Anatomy, Silchar Medical College, Silchar, in Cachar District of Assam, India. The institutional ethical committee approved this study protocol (approval number: SMC/EC/2022/13793). One hundred students who took admission into the 1st year MBBS course for the 2021-22 academic session and met the criteria of having at least 75% attendance in histology were included in the study. They were of similar average intelligence and aged between 18 and 20 years (p = 0.682). Students who did not provide consent and repeaters were excluded from the study. Participants were informed and explained in detail about the objectives and procedures of the study, and informed consent was obtained from them. Among the 100 participating students, 42 (42%) were females and 58 (58 %) were males, and they were assigned equally into two groups: TM group (n = 50) and VM group (n = 50). Group allocation was done by stratified random sampling so that 50% of females (n = 21) and males (n = 29) were included in the TM group, and the remaining unselected participants represented the VM group. All participants had previously used traditional microscopes in their practical classes.

A one-day intervention comprising two events was conducted on a chosen date, and all 100 allocated students participated in the same. The first event of the intervention schedule was a training session on histology, after which the second event of the evaluation process ensued. To execute these events, the “histology of the cardiovascular system" had been chosen unanimously as the topic of our study from the histology syllabus of phase 1 MBBS. For the first event, at the lecture hall, a two-hour-long didactic lecture was delivered by an experienced lecturer and expert in histology, followed by a practical contact session of the same duration carried out through TM and VM for the respective groups. Students were briefed about the practical session and directed afterwards to their designated laboratories on the basis of group allocations, i.e., TM or VM group, to attend respective sessions. Before attending the sessions, students were not aware of their group allocation.

For the study, we selected the departmental best glass slides of the cardiovascular tissues. Glass slides were then digitized by scanning and photographing them both at high (40x) and low (10x) magnification by a slide scanner. For each glass slide, images of multiple fields were generated and captured. Scanned images were then uploaded to Google Drive after storing them on the computer's hard disc, where they could be viewed by a Panoramic viewer software. VM group students (n = 50), before attending the practical session, were instructed about the modus operandi of the virtual microscope. Students were allowed to use their laptops, where we transferred the virtual images to be viewed under VM software so that they could easily toggle around the images stored on the computer's hard disc and Google Drive.

We ensured constant strong internet connectivity during the session. Moreover, for convenience, we also used a binocular light microscope attached to an LCD projector to live-cast glass slide images on the screen. On the other hand, the TM group (n = 50) studied the histology lessons by participating in the TM session with glass slides and light microscopes. The glass slides chosen for the TM session were the same slides used in the VM session after digitization. As the total number of slides was insufficient to ensure a one-to-one ratio between students and glass slides, we asked students to share them among themselves. As in the VM session, various fields of the same slide were focused under a light microscope for optimal visualization and followed afterwards by a thorough discussion with students for proper understanding of histological structures. During the practical session, an expert histology teacher accompanied both groups and guided and trained the learners of the respective groups.

The students were then reassembled at the lecture hall for the second event of the intervention schedule, i.e., the evaluation process that included histological tests and a post-test prepared questionnaire. For the histological test, we conducted theory and practical assessments in the form of multiple choice questions (MCQs) and objective structured practical examination (OSPE), respectively, and subsequently, we distributed a questionnaire to students of both groups regarding the method, either TM or VM, used for teaching and learning.

Both the groups were asked to solve an exact five sets of MCQs, designated as Q SET 1 - 5, each set carried six marks and had six questions, highlighting histology of any of following five tissues: (1) artery; (2) vein; (3) capillary; (4) lymphatic or (5) the heart. The answer key for the MCQs was validated from standard textbooks. The time allotted for the MCQs exam was 30 minutes. After completion of the MCQs test, students were asked to visit their respective laboratories according to their group allocations, either TM or VM, to appear for the OSPE test, consisting of five questions carrying four marks each, that tested their histology diagnosis abilities. OSPE had five test stations and one rest station. The time allotted for each test station, marked by a practical-based question, was three minutes. To perform OSPE test on the TM group, participants had to answer questions related to the glass slides focused on two or three microscopes for each station whereas for performing VM group OSPE test, in each station, participants needed to scroll over several snapshots of virtual slides presented on a desktop and to answer the respective questions.

The MCQs and OSPE tests were supervised by eight staff members, and the students were spread out 2 meters apart while attending them. After completion of the tests, students were given a questionnaire on the learning method, i.e., either TM or VM, that mainly focused on overall understanding of the subject, acceptance rate, convenience of learning, usefulness for productive discussion, and effectiveness as a learning tool. The questionnaire comprised the following six items: (Q1) the operation method was convenient to use; (Q2) the instruction was knowledgeable and helped in better understanding of histology; (Q3) this microscopy provided clearer images; (Q4) this teaching method kept me highly engaged; (Q5) this teaching method was preferable for learning histology; and (Q6) this teaching method can stimulate my interest in learning histology and I want to learn more with this method. The responses for the above items were measured with a 5-point Likert scale (1: strongly disagree; 2: disagree; 3: neutral; 4: agree; and 5: strongly agree).

The comparison of students’ performance and learning skills through TM and VM and their acceptance for the same was carried out through a Student’s unpaired t-test. The data were analyzed by using SPSS Statistics (IBM Corp., Armonk, NY) and Microsoft Excel software. Statistical significance was set at p<0.05 with a confidence interval (CI) of 95%.

## Results

In our study, we evaluated the impact of virtual slides and glass slides on students’ ability to learn histology by conducting histological tests. Virtual slides, prepared by digitizing traditional glass slides, were used as a new form of learning tool. We also assessed students’ acceptance rate for VM and TM through a questionnaire. We recorded more positive responses for VM and higher performance by VM students in histological tests.

**Figure 1:**
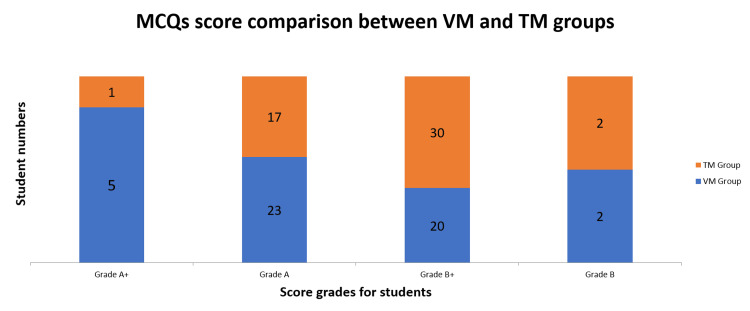
The scores of students’ histological MCQ tests after histology lessons using either VM or TM MCQ: multiple-choice question; TM: traditional microscopy; VM: virtual microscopy

MCQ test results are shown in Table 1 and Figure 1. VM group students who learnt using digitized slides, were quite excellent in MCQ tests as 28 students (56%) from VM group (n = 50) secured A grade (score ≥61%), which was further subdivided into two subgrades designated as “grade A and grade A+”, in comparison to only 18 students (36%) from TM group (n = 50) that used glass slides during training. Five Students (10%) from the VM group obtained grade A+ (score ≥81%); in contrast, only one (2%) student from the TM group reached that milestone. A total of 54 students fell into grade B (score 21% - 60%), whereas 20 (40%) and 30 (60%) students, respectively, from the VM and TM groups, obtained grade B+ (score 41% - 60%) (Figure 1).

**Table 1:** Mean scores for five sets of histology MCQ test questions obtained by students attending either TM or VM learning MCQ: multiple-choice question; SD: standard deviation; TM: traditional microscopy; VM: virtual microscopy

Questions	Mean ± SD		P-value
	VM group	TM group	
Q SET 1	3.98 ± 1.28	3.56 ± 0.76	0.0488
Q SET 2	3.94 ± 1.15	3.54 ± 0.73	0.0405
Q SET 3	3.94 ± 1.05	3.50 ± 0.76	0.0183
Q SET 4	3.90 ± 1.11	3.48 ± 0.88	0.0386
Q SET 5	3.86 ± 1.01	3.46 ± 0.99	0.0483
Mean score	19.62 ± 4.22	17.54 ± 2.93	0.0051

Virtual slides might have provided learners with a clearer concept, enough to overshadow the difficulties associated with glass slides. The difference between the two groups of students’ MCQ scores for all the question sets (Q SET 1 - 5) was statistically significant (p<0.05, Table 1). Besides, results indicating statistically significant differences (p<0.05, Table 1) of mean MCQ scores of 19.62 ± 4.22 for the VM group and 17.54 ± 2.93 for the TM group were also noted.

**Figure 2:**
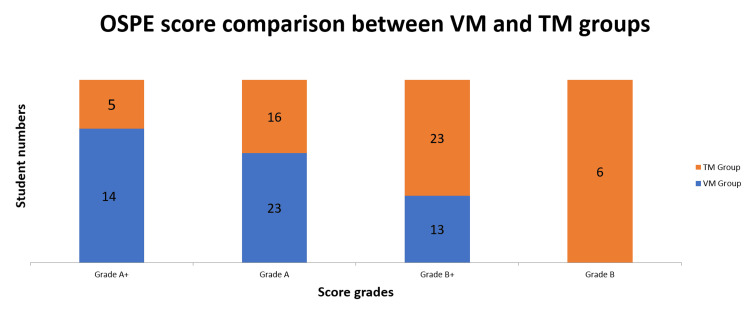
The scores of students’ histological OSPE test after histology lessons using either VM or TM OSPE: objective structured practical examination; TM: traditional microscopy; VM: virtual microscopy

OSPE results are shown in Table 2 and Figure 2. Of note, 37 students (74%) from the VM group (n = 50) obtained an A grade (score ≥61%), and of them, 14 (28%) achieved a grade A+ (score ≥81%). However, from the TM group (n = 50), only 21 students (42%) secured an A grade, while only five students (10%) achieved grade A+. A total of 42 students obtained a B grade (score 21% - 60%), whereas 13 (26%) and 23 (46%) students, respectively from VM and TM groups, obtained a grade B+ (score 41% - 60%). No student from the VM group got a grade B (score 21% - 40%) (Figure 2); however, six (12%) students from the TM group had a grade B score (Figure 2).

**Table 2:** Mean scores for five questions in OSPE test obtained by students attending either TM or VM learning OSPE: objective structured practical examination; SD: standard deviation; TM: traditional microscopy; VM: virtual microscopy

Questions	Mean ± SD		P-value
	VM group	TM group	
Q1	3.48 ± 0.83	2.98 ± 1.15	0.0143
Q2	3.26 ± 0.94	2.68 ± 0.91	0.0023
Q3	2.92 ± 0.94	2.36 ± 1.00	0.0048
Q4	2.72 ± 0.90	2.20 ± 1.17	0.0144
Q5	2.34 ± 0.82	1.90 ± 1.16	0.0309
Mean score	14.72 ± 2.33	12.12 ± 2.97	< 0.001

When we compared the scores of the five OSPE questions (Q1 - Q5), statistically significant (p<0.05, Table 2) differences were recorded, with higher performance pointing more towards the VM group. Mean scores of 14.72 ± 2.33 (73.6%) and 12.12 ± 2.97 (60.6%), respectively, from VM and TM groups were also analyzed, compared, and found to be statistically significant (p<0.001, Table 2). These observations could easily lead us to the assumption of having a powerful impact by VM-induced learning in the practical field of a histology course. VM learning might have improved the understanding of the subject matter by engaging students in active participation, providing them with thorough content, and eliciting thoughtful responses by bridging the distance between technology and learning.

**Figure 3:**
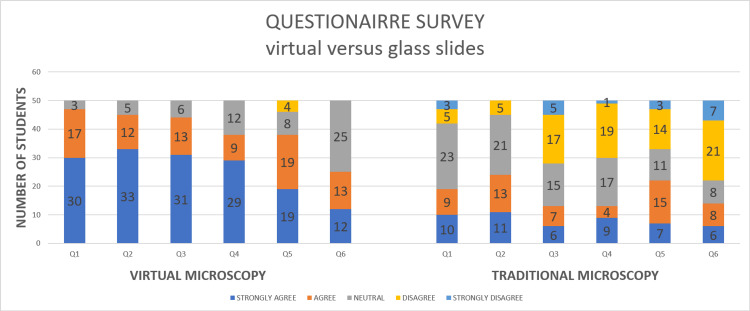
The number of students who answered the six items in the questionnaire after finishing practical histology lessons using either digitized virtual slides or traditional glass slides

Results of the questionnaire are shown in Table 3 and Figure 3. Of note, 79% (237/300) of VM students who used digitized slides for learning agreed and endorsed (agreed and strongly agreed) with the new method, and 51.33% (154/300) of students strongly agreed with VM. On the contrary, only 35% (105/300) of students who applied glass slides for learning agreed and accepted TM, and 16.33% (49/300) students out of them strongly agreed in terms of the efficacy of TM (Figure 3). Most students seemed to be inclined towards using virtual digitized slides rather than undergoing training with a conventional approach by adopting real light microscopy and handling of traditional glass slides. Mean differences of the scores of all six items (Q1 - Q6) of the questionnaire were also found to be significant on statistical analysis (all p<0.001, Table 3).

**Table 3:** The mean acceptance rates of six items in the questionnaire regarding the learning of practical histology using either virtual or glass slides SD: standard deviation; TM: traditional microscopy; VM: virtual microscopy

Questions	Mean ± SD		P-value
	VM group	TM group	
Q1	4.54 ± 0.61	3.36 ± 1.10	< 0.0001
Q2	4.56 ± 0.67	3.60 ± 0.94	< 0.0001
Q3	4.50 ± 0.70	2.84 ± 1.16	< 0.0001
Q4	4.34 ± 0.84	3.02 ± 1.13	< 0.0001
Q5	4.06 ± 0.93	3.18 ± 1.17	< 0.0001
Q6	3.74 ± 0.82	2.70 ± 1.24	< 0.0001

Therefore, after analyzing the results, we concluded that most students preferred learning with the modern method using digitised slides rather than through a light microscope. Test results were also in favor of this statement, as VM learners excel in both theory (MCQs) and practical (OSPE) assessments. Moreover, during VM training, active involvement from participants and establishment of higher interaction among them were also observed.

## Discussion

In this study, we compared the effectiveness of TM and VM in the teaching of histology by conducting MCQ and OSPE exams among medical students, and also distributed a learning method feedback questionnaire to further elaborate on the findings. Most of the previous studies on VM were focused only on students’ perception via questionnaires, by evaluating theory or practical lessons, or both, in the context of VM evaluation. We added more depth to the VM study protocol by including both a questionnaire and a histology test. Our study is the first of its kind to be conducted in a medical college in Assam. To the best of our knowledge, in recent times, no similar study like ours has been conducted in Northeast India.

The shortcomings associated with TM limit the learning process of histology. Our study was designed to overcome these drawbacks by employing the VM system in the histology class to evaluate its efficiency from the students’ viewpoint. We found overall higher performance trends in favor of VM. Students of the VM group had higher mean test scores both in MCQs and OSPE. The mean acceptance rate was also higher for VM than TM, as evaluated by the questionnaire interpretations. Similar results were also obtained by Evans et al. [28] in their study, whilst aiming to determine the effectiveness of VM over TM in microscopy teaching concerning its eventual application. They found that VM outperformed the TM group significantly in a practical histology assessment. The mean assessment score of the VM group (14.18, 49.75%) was significantly higher than the TM group (12.77, 44.80%). This difference in score was statistically significant (p = 0.048), as in our study, where we observed significant differences (p<0.001) between mean OSPE scores of VM (14.72, 73.60%) and TM (12.12, 60.60%).

For the query, “Whether the method is convenient, i.e., easy to find good areas of the slide”, they recorded values of 4.51 and 4.05 (p = 0.003) for VM and TM groups. respectively, whereas in our study, the values we obtained were 4.54 (VM) and 3.36 (TM) with a p-value <0.001. Furthermore, the difference in mean score for the item, “whether they were highly engaged by the method”, in their study (VM = 4.42, TM = 4.38), was not statistically significant (p = 0.511). However, we found that (VM = 4.34, TM = 3.02) to be significant (p<0.001). On a similar note, Wu and Chiang [26] also documented a higher rating for VM among students when they recorded that 84.78% of students (79% in the present study) accepted and agreed with VM learning in comparison to only 34.06% (35% in the present study) for TM. Besides, students undergoing training with VM learning possessed higher histology diagnosis abilities, and hence they concluded that VM is a great method of learning histology, and it may gradually replace TM for teaching the histology laboratory course.

The convenience or ease of use of VM was well documented in our study, and this postulation is further strengthened by the study results of Sağol et al. [29], where students positively rated VM, particularly in the context of its user-friendly interface, superior quality images, and improved understanding through collaborative learning. In the present study, we also documented students' finding improvement in the clarity of microscopic tissue images, which has always been a major problem associated with TM. Similar views were also provided by Szymas and Lundin [30], who mentioned that students rated VM as a didactic tool over 8-9 on a 10-point scale, image quality as very good, as well as rated VM as highly preferable over other methods.

In our study, students considered VM instruction as valuable, and it helped in better understanding and learning histology in comparison to TM. Correspondingly, Mondal et al. [31] reported similar results when they compared the TM and VM approaches of histology teaching via questionnaires to dental students following a thorough discussion of topics through both TM and VM. Their study result favored VM in terms of level of understanding, clarity, effective communication, and discussions. Sahota et al. [32] made similar observations in their study where whole slide images were shared with students, and their performance was seen to be improved on analyzing by online quizzes, signifying the positive benefit gained by students through the WSI system. This also aligns with the results obtained by Wu and Chiang [26], wherein 18 out of 23 students rated VM to be helpful to learn oral histology as opposed to 10 out of 23 for TM (p<0.05).

Correspondingly, Francis et al. [33] postulated VM to be a good adjunct to TM with regard to the revised medical curriculum. In their study, higher grades were obtained by VM students than their TM counterparts, both in spotter (practical) and theory tests (which assessed histology knowledge and understanding), as well as documentation of higher positive responses for VM. The analysis of feedback from students is further supportive of our study results. Moreover, Lee et al. [34] investigated the influence of VM on academic performance while comparing laboratory test scores before and after its implementation and documented that the VM group had fewer failing grades, higher test scores, and also showed less laboratory test score variability than the TM group. They also documented that VM enhanced laboratory learning and might have helped students in understanding microscopic laboratory content better.

Intrinsic motivation is key to learning, and by reinforcing the same, better learning and achievement among students can be ensured. We found that the majority of students believed VM stimulated their learning so that they wanted to learn more with VM and this was evident from their higher mean score of 3.74 (2.70 for TM, p<0.001) for the relevant item in the questionnaire, “This teaching method can stimulate my interest in learning histology and I want to learn more with this method”. This is in partial alignment with the survey by Simok et al. [11], who evaluated the influence of VM on intrinsic motivation among medical students and found “perceived competence”, an attribute of intrinsic motivation, to be higher in the VM group.

This is also in alignment with the students' score results to the queries posed by Wu and Chiang [26] who recorded a scored 3.78 (p<0.05) for the query, “whether VM stimulated histology learning”, and Evans et al. [28] who obtained a mean score of 3.77 (p<0.05) for the item, “I will learn more with VM”) when enquired about its ability to stimulate a sense of learning among students and whether they wanted to learn more with it. This finding is also in concordance with the findings of the study by Qing et al. [14], where students not only positively rated VM but also believed that it was associated with increased learning focus and development of independent thinking, which further indicated their overall satisfaction with this system. Moreover, the average score of the final laboratory exam also improved from 62% before to 83% after the implementation of VM in their study.

To explore the teaching and learning conditions that would better suit VM for enhanced outcomes, Herodotou et al. [35] compared VM learning in blended and online-only modes through data collected via surveys and interviews of medical students. Their research revealed equally good perceived learning gains by both blended and online-only modes in students, but the former approach is better in terms of student engagement and satisfaction. As in our study, examining students’ attitudes on all the subjective impressions related to convenience, suitability, preferability, or satisfaction, they also revealed similar high-scoring patterns among students of VM than those of TM. This result is also in keeping with the previous study by Santos et al. [36] who also noted significant differences between the scores of VM and TM students with regard to the above-mentioned impressions; however, in the context of comparing academic performance, these results were insignificant (p = 0.38) and that contrasts with our study (mean OSPE score p<0.001).

Likewise, Amer et al. [37] did not observe any significant differences in scores related to VM versus TM learning when VM was used for online OSPE assessment of students to evaluate its efficiency in improving medical students’ histology scores. Nonetheless, online surveying on VM revealed positive feedback from students, and net satisfaction on all items ranged from 3.70 to 4.25 on a Likert scale, which is close to our study findings (3.74 to 4.56 mean satisfaction scores). Correspondingly, Koch et al. [38] in their study, when comparing diagnostic accuracy and efficiency between VM and TM, did not find any statistically significant difference between correct answers by residents regarding dermatopathological disorders. Although most residents supported the use of VM as a learning tool, a significant number of residents reported difficulties with it, like fuzzy images, poor screen color, freezing of the screen, poor screen contrast, imaging software problems, computer failure, power failure, and issues with adjusting the computer monitor. VM has its disadvantages, including the neglect of TM use and encountering of frequent technical difficulties [22]. Use of VM also warrants good internet connectivity, enormous computer memory storage, and ensuring students’ off-campus accessibility to VM [24]. However, off-campus availability of VM slides cannot be guaranteed for every student. Slow internet connection and frequent technical errors are some other major issues linked with VM.

Moreover, the VM system could be delivered through an online mode, which explains its utility in histology teaching during a pandemic like COVID-19, when medical classes are switched to online mode. Besides, Collier et al. [39] analyzed instructor evaluation by conducting interviews of teacher assistants, and analyses of instructor interviews showed that the positives of VM use in an undergraduate anatomy course outweigh the potential negatives. They cited convenience and accessibility of VM use as well as increased student collaboration as key advantages of VM. Correspondingly, Ordi et al. [40] postulated about the effective replacement of TM with VM to teach pathology in medical schools, and microscopic skills acquired with digitized slides are comparable to those acquired with glass slides. The augmentation of conventional microscopy with virtual microscopy shows an enhancement of the understanding of the subject than when they are used as a standalone modality [8]. Moreover, VM is economical and also offers affordable maintenance, while positive values of TM-based learning are still retained [41]. By providing a virtual platform for student and teacher interaction, VM offers an inclusive environment, particularly for shy students who struggle with face-to-face communication [16].

When the multimedia tools are integrated with the learning process, students find the learning topics more interesting [42], and the learning process is also significantly affected by the presentation mode of learning content [43]. Most of today’s generation of students grew up with the internet and are good at rapidly adapting to it; they can maximize the utilization and exploration of VM learning [14]. They are eager for pragmatic and convenient learning, and also technically skilled for participating in online learning platforms [44]. As they exhibit the propensity for VM-like technology and it might also be suited to their preferred style of learning that favors assistance from computers and the internet. As learning style is one of the most important components of the learning process, understanding it better might help figure out an optimal study duration for lessons to enhance academic achievements [45]. Preferred learning styles also help students to recognize their strengths and weaknesses and increase their learning potential [46]. We can assume that most students rated the VM learning system as the preferred method because it is a modern technology-based system that seems to be perfectly suitable for the learning style of Generation Z, who is characterized as being tech-savvy and independent. 

Although VM has all the potential to completely replace TM, the importance of TM could not be ignored as students develop thought processes and learning strategies while handling real specimens with light microscopes. Moreover, active engagement of students is still being highly developed via physical interaction with a microscope, even in the context of limited visual learning dimensions of TM [47]. The TM handling procedure is more authentic, searching for structures under a microscope is more explicit due to the lack of a permanent overview image, and student-to-student interaction is also achievable as they operate through different individual sections of the same paraffin-embedded tissue [48]. Furthermore, in the medical curriculum, the attainment of light microscope skills by students is desirable, and they should be able to acquire adequate expertise on using the light microscope as the course progresses [41]. Additionally, physicians require the skill to operate an optical microscope, if not for histology, then at least for other basic professional tasks like gram staining and examination of urine or blood smears [6].

Nevertheless, the goal of microscopic anatomy is not about using the microscope but to teach students normal and abnormal human structure [49]. Rather than limiting the task to manipulation skills, we must be focused more on the evaluation of VM's impact on histology knowledge acquisition [48]. Although VM retains some of the currently existing issues, we believe it to be highly useful for histology learning. To fully realize the benefits of VM, we recommend the use of VM along with TM. This will also be in accordance with the current popular trends and attitudes that advocate the blending of VM and TM approaches. Virtual microscopes allow students to refer to and discuss the exact point on a slide, as students can view precisely the same field of the specimen, thus improving collaborative learning. Collaborative learning improves the needed social skills for the execution of future professional work, besides having cognitive benefits like improved academic performance, motivation, and so on [50]. Thus, integration of VM with the current education system, as per our experience, will help devise an important new modality and modern approach to histology teaching and fully justify its addition as an important learning platform for the histology course. It will bridge the knowledge gap among students and provide the convenience of learning, ultimate accessibility to good-quality images for study, and many other advantages of VM with TM.

This study has certain limitations. The study involved a relatively small sample size, consisting only of first-year MBBS students. Involvement of first-year BDS students and second-year MBBS pathology students could have brought in more diverse and interesting perspectives to the study. Moreover, the duration of the study was short, and the material taught was relatively non-explicit. Hence, we recommend more extensive, exclusive, and comprehensive studies to gain deeper insights into the topic.

## Conclusions

VM is highly useful in the teaching of histology and superior to TM in various ways, mandating regular use of VM in histology classes as part of advanced learning, particularly in medical colleges in places like Assam, where TM remains the preferred method of histology teaching. As students performed better under the VM system, it may be practical to incorporate the same as a mainstay in the histology laboratory, and the integration of virtual microscopic digitized slides into the framework of histology teaching warrants serious further attention in the future. As our study is the first of its kind in medical colleges in Assam, we believe it will pave new ways in the field of histology learning for other researchers to undertake further studies and keep up with the modern medical curriculum that is constantly evolving and demands the extensive use of technology.
